# Fast Wearable Sensor–Based Foot–Ground Contact Phase Classification Using a Convolutional Neural Network with Sliding-Window Label Overlapping

**DOI:** 10.3390/s20174996

**Published:** 2020-09-03

**Authors:** Haneul Jeon, Sang Lae Kim, Soyeon Kim, Donghun Lee

**Affiliations:** School of Mechanical Engineering, Soongsil University, Seoul 06978, Korea; skyjeon95@gmail.com (H.J.); sanglaekim37@gmail.com (S.L.K.); sue.kim163@gmail.com (S.K.)

**Keywords:** time-series data, biomechanics, walking gait, CNN, sliding window, wearable sensor

## Abstract

Classification of foot–ground contact phases, as well as the swing phase is essential in biomechanics domains where lower-limb motion analysis is required; this analysis is used for lower-limb rehabilitation, walking gait analysis and improvement, and exoskeleton motion capture. In this study, sliding-window label overlapping of time-series wearable motion data in training dataset acquisition is proposed to accurately detect foot–ground contact phases, which are composed of 3 sub-phases as well as the swing phase, at a frequency of 100 Hz with a convolutional neural network (CNN) architecture. We not only succeeded in developing a real-time CNN model for learning and obtaining a test accuracy of 99.8% or higher, but also confirmed that its validation accuracy was close to 85%.

## 1. Introduction

Human motion recognition (HMR) is a technology domain that recognizes and distinguishes different types of human activities using sensor data [1]; it is widely used in rehabilitation and medical treatment like the classification and rehabilitation evaluation of patients with hip osteoarthritis, neurological disorders such as stroke, and Parkinson’s disease through gait analysis [2,3,4,5,6,7,8,9,10,11]. It also has been used in training assistance like exercise coaching through motion tracking and feedback, speed and position tracking in sports training [12,13,14,15,16,17], sudden fall prevention [18] along with the development of wearable sensor technology. This paper describes the development of a foot–ground contact phase classification (FGCC) algorithm as FGCC is one of the most fundamental and elemental processes in lower-limb motion analysis.

The major sensors used in HMR research can be categorized as cameras, force sensors, and inertial motion sensors. According to Farooq et al. [19], 20 human motions were classified with 74.4% accuracy using an RGB-Depth camera. However, in order to obtain an acceptable quality of three-dimensional (3D) point cloud data (PCD) of the entire human body, which is necessary for algorithm training, the camera must be accurately aligned with the coronal or frontal plane of the human body; noise such as outliers in the depth map must be eliminated as well. Abellanas et al. [11] and Kim et al. [20] achieved 99.8% and 93.1% accuracy in foot–ground contact detection with force plates and force sensitive resistors (FSRs), respectively. However, as their methods were based on measuring the physical contact between the foot and the ground, lower-limb motion could not be analyzed simultaneously. Qui et al. [7] and Mohammad et al. [9] proposed a method for the independent detection of walking, squatting, and jumping using wearable inertial sensors. Although a wearable inertial sensor is very easy to use and has limitless measurement workspace [21], acceptable detecting accuracy has not been continuously obtained owing to sensor drifts as well as initial calibration issues [22].

In HMR algorithms, rule-based applications for class prediction based on a threaded range of feature data extracted through sensor data analysis as well as various neural network paradigms, such as convolutional neural network (CNN) and region-based CNN (R-CNN) have been utilized. In the study by Kim et al. [23] and Teufl et al. [24], a rule-based classifier with 99% accuracy was developed after examining the major features of the foot-to-ground contact phase classification through data-driven analysis. Shin et al. [1] developed an inertial and altitude sensor data based human activity classifier with a long-short term memory (LSTM) architecture. The model was able to classify six static gestures with 99.92% classification accuracy. Similarly, Hsu et al. [25] applied principal component analysis (PCA) and support vector machine (SVM) for the classification of 10 different routine activities and 11 dynamic activities. They achieved classification accuracies of 98.23% and 99.55% for routine and dynamic activities, respectively. However, they discussed the limitation of their model in that the accuracy was greatly affected by individual datasets and the number of routine and dynamic activities. The study conducted by Janidarmian et al. [26] classified activities using 293 different machine learning algorithms. Their work used PCA to identify the features in the data from 70 activities in real environments allowing for the fact that a wearable acceleration sensor’s attachment position, posture, and learning algorithm affects the performance of the recognition model. As a result, it was suggested that the human activity of all subjects could be recognized with an average accuracy of 96.44% through the K-fold evaluation method, and that human activity recognition could be performed with an average accuracy of 79.92% for each subject. To improve this, Almaslukh [27] proposed a method that could recognize human activity in real-time while not being affected by the location of the attachment. Using RealWorld Human Activity Recognition (HAR) public data [28], a hyper-parameter tuning was performed for optimal learning on CNN and eight dynamic activities were classified with 84–88% accuracy. In the study conducted by Um et al. [29], 50 upper-limb resistance exercise movements were recognized with 92.1% accuracy by a CNN unrelated to the sequence of time, instead of by a recurrent neural network suitable for time series data. The data set they used was the time-series data of an inertial sensor, which was given by the PUSH Sensor Company; the time-series data could be imaged through the sliding window method to learn the classification model on CNN. However, as 99% of the exercises ended within 3.92 s, input image format was performed within 3.92 s for all exercises, which led to a limit in recognition in real-time owing to an inability to distinguish various phases that make up one action, such as the foot–ground contact phase and the swing phase.

In this study, based on a lower-limb wearable inertial sensor and CNN model, an FGCC algorithm is developed that can recognize the four phases of heel strike (HS), full contact (FC), heel off (HO), and swing (SW) in real time. In order to distinguish the multiple phases in real time in a very short time interval based on time-series data of lower-limb behavior collected by inertia sensors, it is most important to secure a labeled time-series motion dataset and convert it to a neural network (NN) input image. Therefore, in this study, a sliding window-based label overlapping (SLO) method is proposed to secure an effective labeled time-series motion dataset. The most significant research contribution of the proposed method is that it makes it possible to obtain a dataset capable of learning a real-time FGCC algorithm with high-recognition precision based on the NN structure without modification of the existing time-series motion data acquisition method. The 13,837 raw time-series datasets collected directly in this study were expanded to 575,880 through data augmentation, then, divided into 60% training sets and 40% test sets; the performance of the proposed method was verified through actual validation experiments.

In this paper, Section 2 defines the research objective; the experimental equipment, data collection, and data labeling are also explained. Section 3 consists of a description of the data preprocessing and application of the SLO method. In Section 4, the model design, selection of optimal parameters using the Taguchi method and validation are described. Finally, in Section 5, the paper concludes with a discussion of the results, limitations of this study, and future research possibilities.

## 2. Foot–Ground Contact Phases and Labeling Method

**Figure 1:**
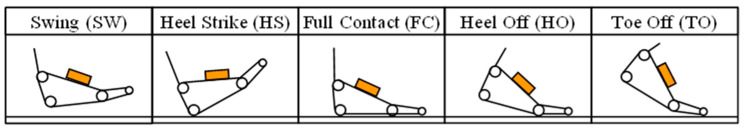
Foot–ground contact phase definition: swing, heel strike, full contact, heel off, and toe off.

In biomechanics and ergonomics, the walking phases are generally divided into a swing and a stance phase, according to the contact between the foot and the ground. As shown in Figure 1, the stance phase, defined as the foot–ground contact phase in this study, can be subdivided into the following four sub-phases: heel strike, full contact, heel off, and toe off [23,30]. As the goal of this study is to accurately and individually detect these multiple sub-phases only with wearable inertial motion sensors on the lower-limb part, an additional measurement device for labeling the sampled inertial motion sensor data according to the sub-phase should be considered in the training dataset acquisition process.

**Figure 2:**
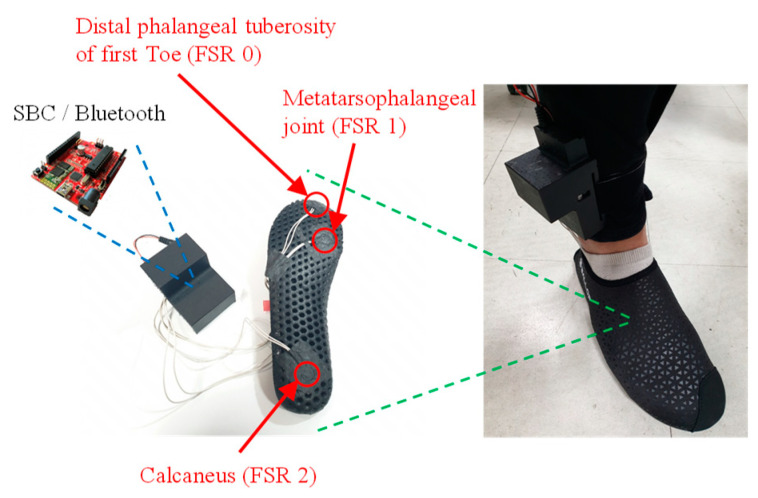
FSR-arrayed insole for dataset labeling of the four sub-phases in the stance phase.

**Figure 3:**
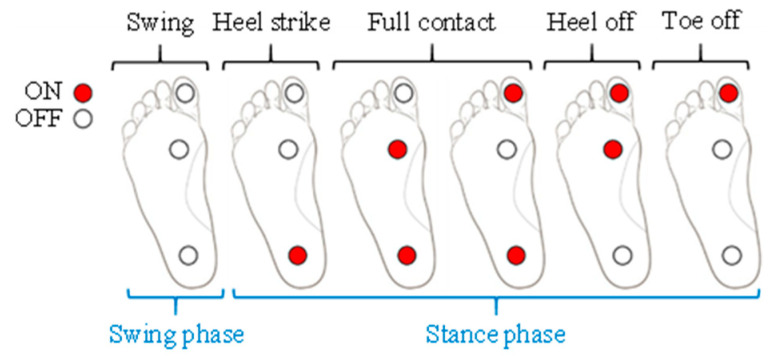
Four sub-phase labeling criteria according to the 3-ch FSR measurement result.

In this study, FSR-arrayed insoles were fabricated, as shown in Figure 2 Considering the foot pressure distributions in each sub-phase, one FSR sensor was attached to each of the three following parts: the distal phalangeal tuberosity of the first toe, the metatarsophalangeal joint, and the calcaneus [31]. A single board computer equipped with Bluetooth modules was also assembled onto the FSR-arrayed insole, as shown in Figure 2, so that the lower-limb motion data acquisition and the data labeling process could be simultaneously achieved without any restrictions on the subjects’ walking range. Figure 3 shows how to determine the individual foot–ground contact phase according to the 3-ch FSR measurement result. (Refer to Appendix A for Pseudocode of four sub-phase labeling process.).

**Figure 4:**
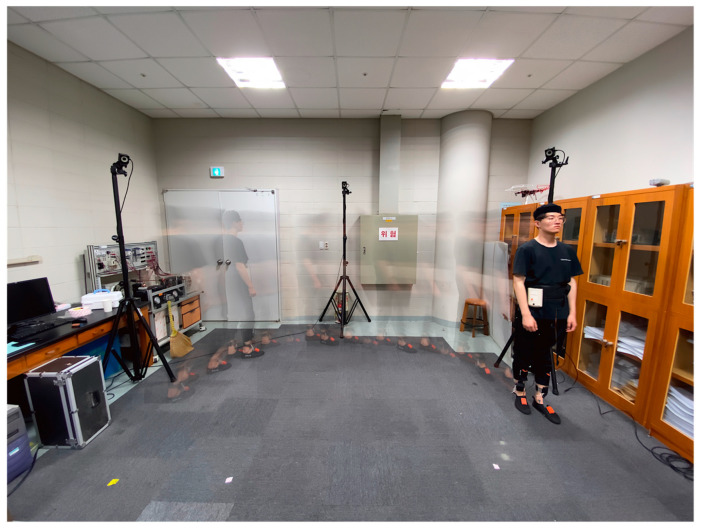
Experiment environment for data acquisition of phase detection in a motion capture area.

**Figure 5:**
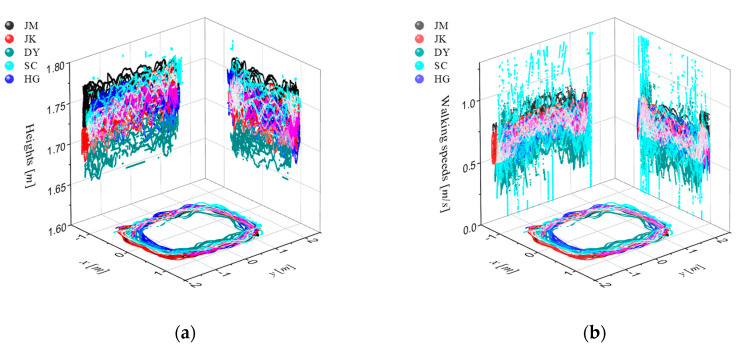
Walking trajectories (**a**) and speeds (**b**) of every subject measured with six OptiTrack Prime 13 cameras.

To examine the feasibility of real-time and individual detection of these four foot–ground contact phases, and to identify how many times each sub-phase is detected in one stance phase while walking at a normal pace, a feasibility study, as shown in Figure 4, has been performed. Thus, a motion capture system was built to track the walking trajectories and speeds with six OptiTrack Prime 13 vision cameras. The number of detections per each sub-phase at various walking speeds and in various directions has been successfully recorded in real time. As a result, as shown in Figure 5, it was confirmed that the walking speed range was 0.2–1.5 m/s, and the walking range was found to be within 3 m × 2 m.

**Figure 6:**
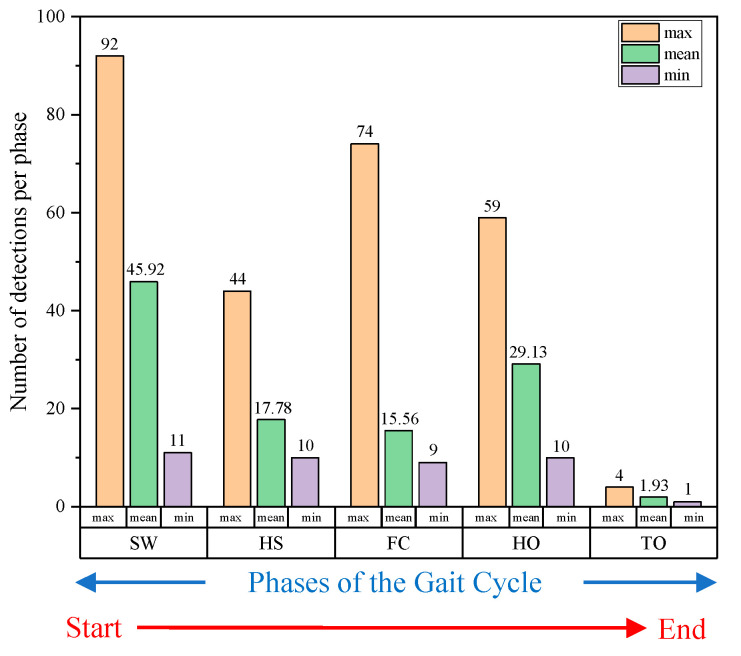
Results of the feasibility study in terms of numbers of the maximum, minimum, and average detections per sub-phase.

Figure 6 shows the results of the performed feasibility study in terms of numbers of the max., min., and average detection per sub-phase. An average of 1.93 toe off phases were detected during one cycle, which is a very short time period corresponding to just 1.59% of a single walk. Because we are going to use the sliding window to extract the wearable motion sensor data, it is expected that the average and minimum number of detections for each phase have a very significant correlation with the sliding window capture width and the FGCC accuracy. This is the reason why we now check the number of detections of each sub-phase per walk prior to determining the class. We will discuss effects of the correlation between these two factors in Section 3 in more detail.

## 3. Data Acquisition and Preprocessing

This section may be divided by subheadings. It should provide a concise and precise description of the experimental results, their interpretation as well as the experimental conclusions that can be drawn.

### 3.1. Training Dataset Acquisition

**Figure 7:**
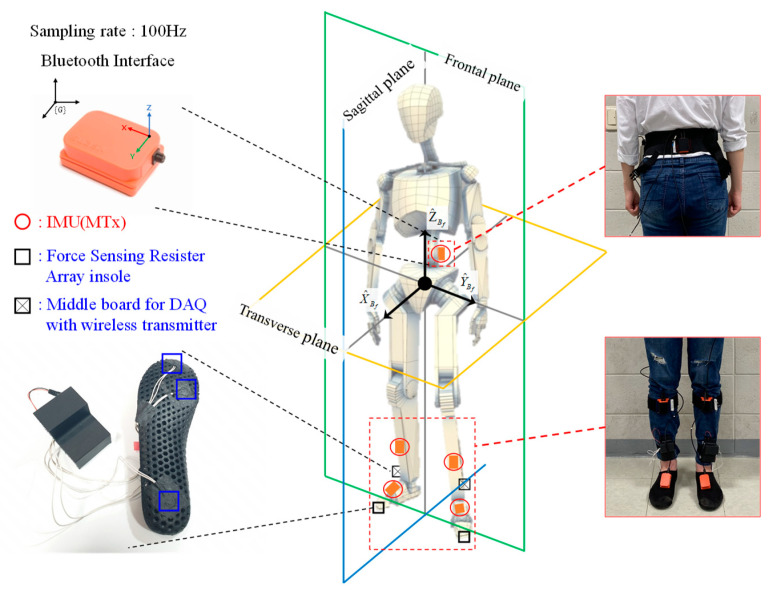
Configuration of the designed wearable experimental equipment for labeled lower-limb walking motion dataset acquisition.

To obtain labeled walking motion data for the lower limbs, wearable experimental equipment was designed with five wireless inertia measurement unit (IMU) sensors [32] attached to each segment of the lower-limb part and a wireless FSR-arrayed insole unit for sub-phase labeling, as shown in Figure 7. An operating console for integrated data collection and preprocessing is installed near the subjects. The IMU sensors, measuring 3-axis orientation, 3-axis angular velocity, and 3-axis acceleration at 100 Hz, were fixed on each foot, shank, and waist using rubber straps.

**Figure 8:**
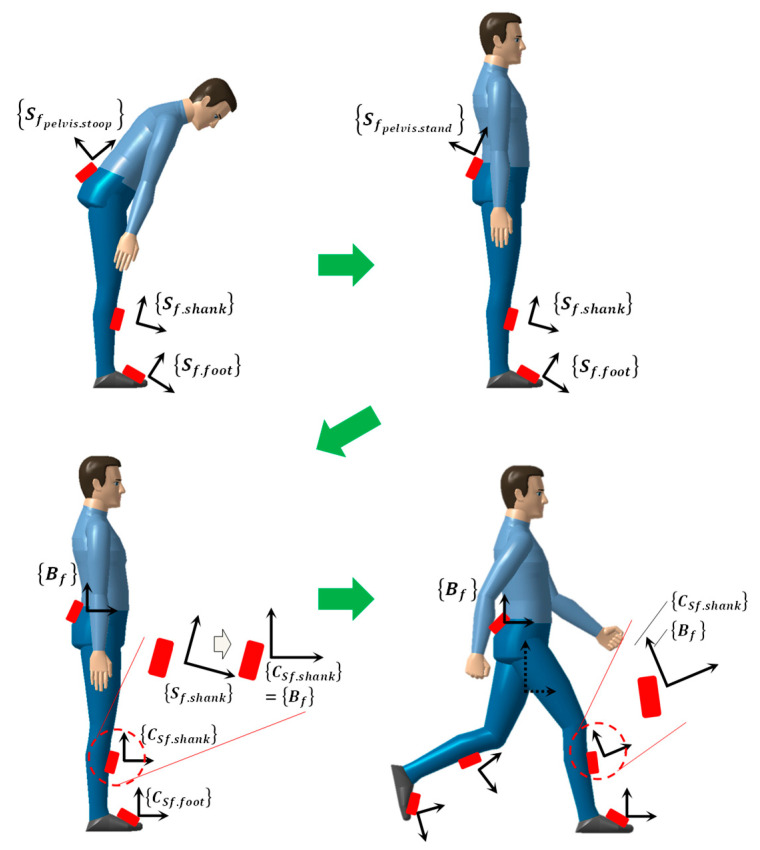
Procedure of standing–stooping calibration motion for creating a common sensor-fixed reference coordinate frame.

Owing to the nature of the wearable sensors, every sensor attached to a lower-limb part has at first different positions and orientations. However, because IMU sensor output is expressed with respect to its own sensor-fixed coordinate frame, we created a common reference coordinate frame by using initial sensor calibration gestures. In this study, the standing–stooping calibration motion, which is the result of our preceding research [23], as shown in Figure 8, was applied.

**Figure 9:**
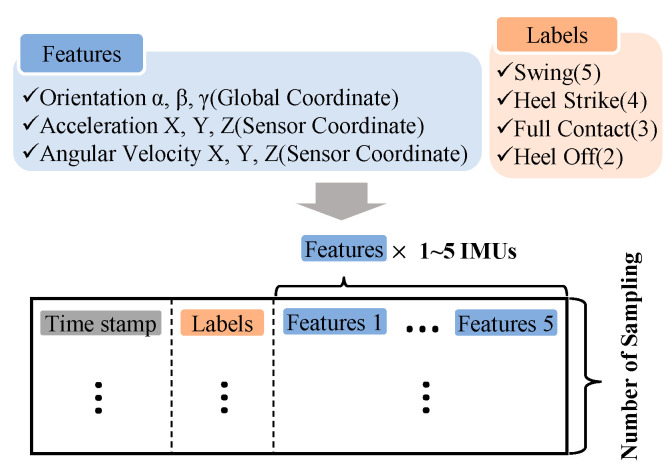
Low-level communication protocol: timestamp, labels, and inertial motion sensor data.

In the operating console, the wearable motion sensor data were integrated with the label from the FSR-arrayed insole as shown in Figure 9. A single integrated message is composed of a timestamp, labels (left FSR, right FSR), and IMU sensor feature data, in that order.

### 3.2. Data Augmentation

**Figure 10:**
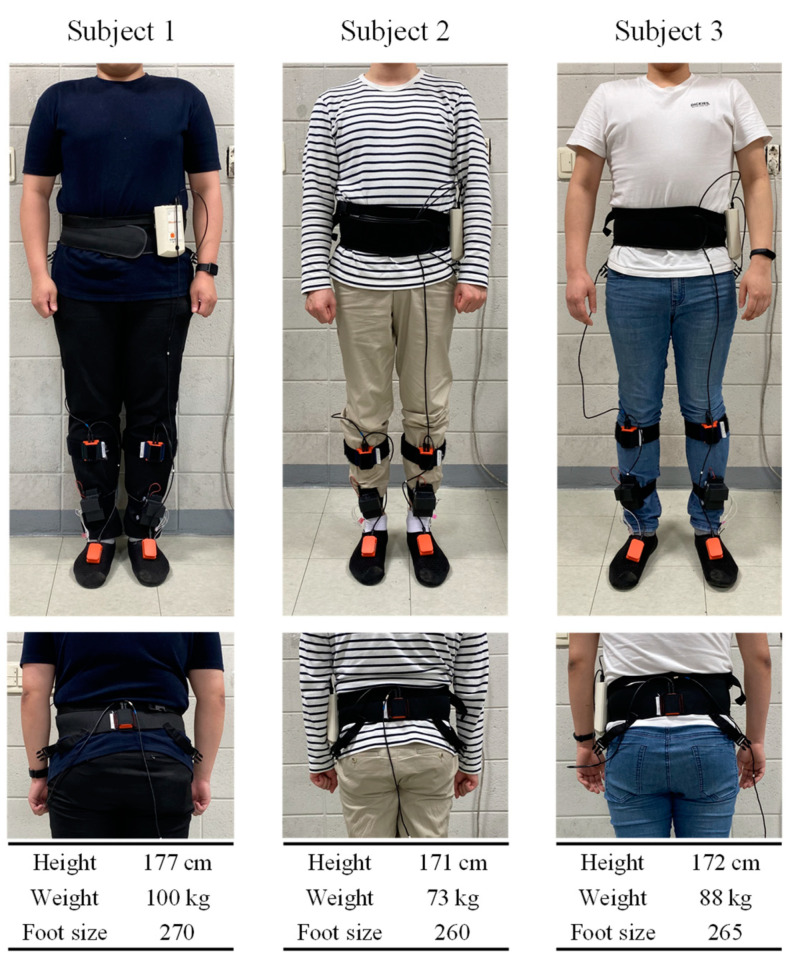
Experimental environment and subject information.

In this study, three subjects, as shown in Figure 10, participated in flat ground walking experiments at a speed of 0.24–1.37 m/s to collect a labeled walking motion dataset of the lower limbs. Around 14,000 raw labeled datapoints were successfully obtained.

In order to significantly improve the generalization accuracy of the trained models without actually collecting new datasets, white noise was added to the entire raw dataset, as in Equation (1):

(1) S[n]±{max(|S[n]|)−|mean|}×0.1=N[n]

where, S[n]denotes the feature data of ${\mathbb{R}}^{n\times 1}$. As a result, the raw data were increased by about 25 times to a total of 359,924 datapoints.

### 3.3. Standardization

**Figure 11:**
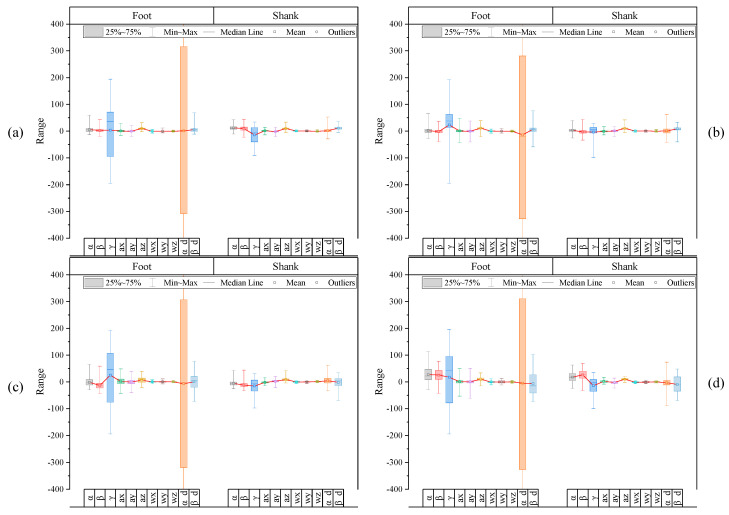
Box plot results of the augmented raw feature dataset with mean, standard deviation for each label, (**a**) label 2, (**b**) label 3, (**c**) label 4, (**d**) label 5.

As shown in Figure 11, because the augmented raw feature dataset still comprises data of various scales, standardization, which is the process of rescaling one or more features so that they have a mean value of 0 and a standard deviation of 1, had to be performed. Let us suppose this standardization process is not performed before training our models. If the distribution of a specific feature data is relatively low compared to the distribution of other feature data, the feature data may possibly be incorrectly evaluated as a feature that does not contribute to improving classification accuracy owing to its relatively low sensitivity in the corresponding class.

This is the reason why we performed standardization according to Equations (2) and (3) on the augmented raw feature dataset. Basically, as standardization assumes that the data has a Gaussian distribution, this process is more effective if the distribution of the feature data is Gaussian. It was confirmed that the distribution of our data does not follow an exact Gaussian distribution, but shows a very similar trend.

(2) $$\sigma_{j}=\sqrt{\frac{\sum_{i=1}^{N}{\left(x_{i,j}-E\left(x_{j}\right)\right)}^{2}}{N}}$$

(3) $${\tilde{x}}_{i,j}=\frac{\left(x_{i,j}-\overset{-}{x}\right)}{\sigma_{j}}$$

where, *N* and *j* denote the total number of the feature dataset and the feature index, respectively. $x_{i,j}$ and $E\left(x_{j}\right)$ represent a feature datapoint and a mean of the *j-*th feature $x_{j}$, respectively. $\sigma_{j}$ and ${\tilde{x}}_{i,j}$ are the standard deviation and standardization results of $x_{j}$, respectively.

**Figure 12:**
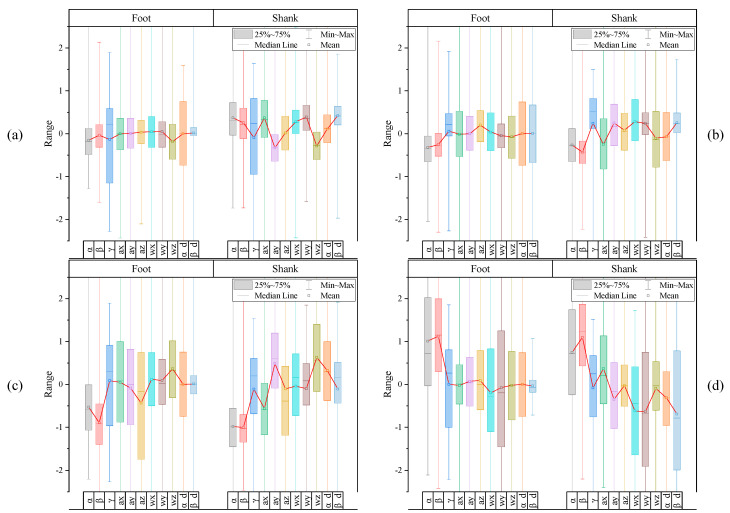
Box plot results of the feature dataset after standardization with mean, standard deviation for each label, (**a**) label 2, (**b**) label 3, (**c**) label 4, (**d**) label 5.

The standardization results through Equations (2) and (3) are shown in Figure 12. It could be confirmed that the relative differences in scale of specific feature data within each label were significantly reduced. In addition, it is expected that the distribution pattern of features between labels will show a distinct difference, which will be a positive factor for multiclass classifier learning. (Refer to Appendix B for mean and standard deviation values for all subjects and Appendix C for mean and standard deviation plots for each subject.)

### 3.4. Sliding-Window Label Overlapping Method

**Figure 13:**
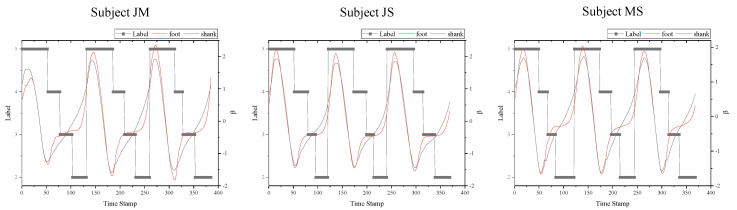
Pitch angles of foot and shank of three subjects.

In our preceding studies, it has been confirmed that the beta angles, also considered as the pitch angle, has significant sensitivities in the foot–ground contact detection [23], as well as some regular changing patterns over time during walking. As shown in Figure 13, the overall patterns of the beta angles of three subjects show quite similar tendencies; the difference in its values according to the label is very significant. Therefore, if a certain period of these feature data with distinct differences according to labels is extracted and converted into an image, class classification may be possible with an NN architecture, such as CNN.

**Figure 14:**
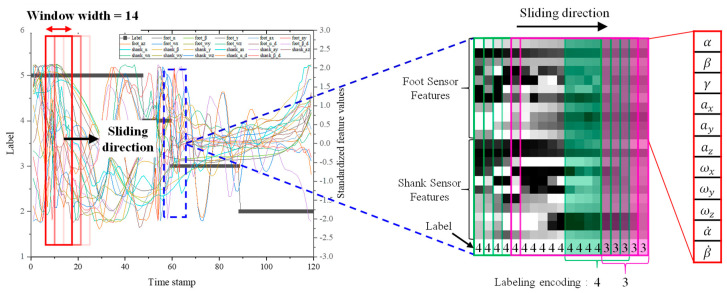
Sliding-window label overlapping method.

Based on this, only the features that could contribute to the FGCC were carefully selected and the corresponding feature data plot over time only includes the right foot and shank motion data, as shown on the left side of Figure 14. Figure 14 shows the entire process of the SLO method. A width of the sliding window can be considered as the desired time span to be extracted of the time-series feature data. If the sliding window width and the sampling frequency are set at 14 and 100 Hz, respectively, a finite-horizon of the sliding window including 14 series of labeled feature data shifts right every 10 ms; hence, the name sliding-window label overlapping. It is important to note that the extracted feature data with SLO of a 22 (height) × 14 (width) window may be mixed with several different labels of the FGCC. As mentioned earlier, because label overlapping must inevitably occur due to the nature of the time-series data obtained in the stance phase, it should be noted that one sliding window may include one to three labels. However, because it is very rare to include three different labels at the same time, a sliding window including three different labels is regarded as an outlier. That is, for the HO phase, most sliding windows including the HO phase are outliers, because the HO phase is detected only 1.93 times on average during one walking step as well as being located between the TO and SW phases. Therefore, HO is integrated into the TO, which has a similar tendency to SW in the phase adjacent to HO. The pseudocode in Appendix D describes how to assign a label in the label-overlapped sliding window and how to convert the window into the image in more detail. The mat2gray function in MATLAB is used to convert the NN data to grayscale image in this study [33].

### 3.5. Validation Dataset Acquisition

**Figure 15:**
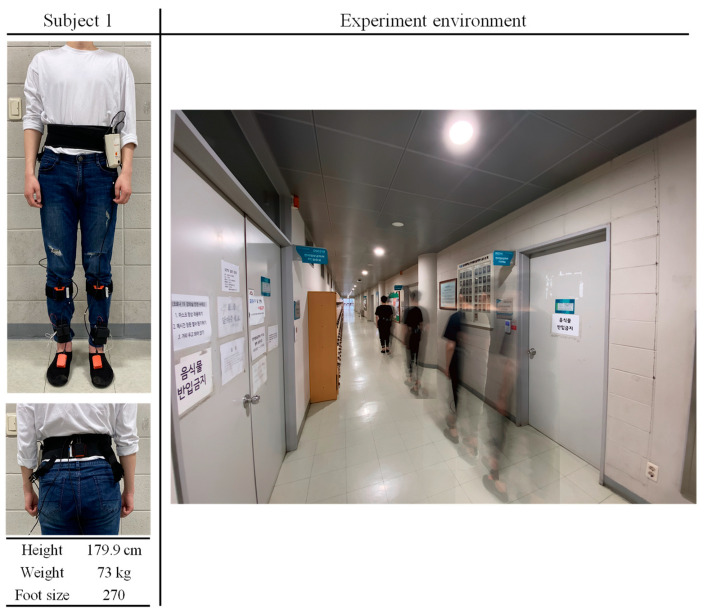
Environment and additional subject for acquisition of the validation dataset.

In addition to the training dataset, the data of new subjects were collected to examine the validity of the trained model as shown in Figure 15. The data were collected on a 49.6 m long flat ground at a speed of 1.21–1.37 m/s; the collected data were preprocessed using the same method except for the data augmentation (refer to Appendix B for standard deviation and mean values of each label) described earlier to generate a total of 1506 efficient validation image sets.

## 4. CNN Model for Real-Time FGCC

**Figure 16:**
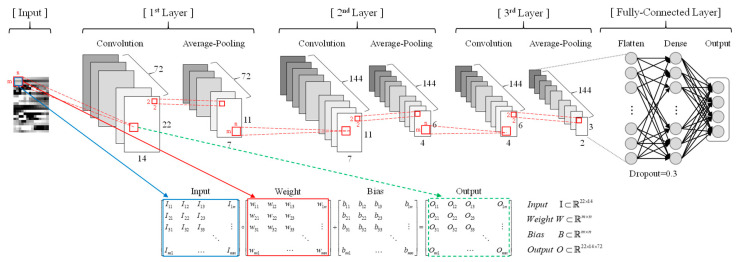
Four label classification CNN architecture.

The structure of CNN in this study was designed as shown in Figure 16. It consists of three consecutive convolutional layers and a fully connected layer at the end. Each convolutional layer performs convolution internally and a pooling process repeatedly to produce various feature maps for the input image. The initial hyper parameters are presented in Table 1, and the learning rate is set to 0.001 as the default value. ReduceLROnPlateau was used to lower the learning rate when the loss did not improve so that the local minimum could be exited. In addition, to prevent overfitting, the callback function EarlyStopping was used to stop learning when the performance of test loss no longer increased.

### Sensitivity Analysis

**Figure 17:**
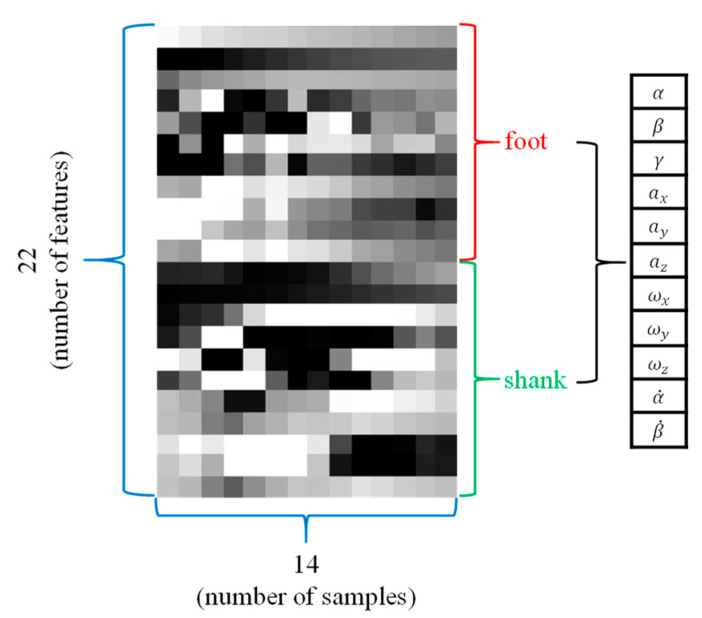
Sample input image.

As CNN was originally developed for image recognition [34], it is important to select the optimal hyper parameter that affects the learning accuracy of the image used. One of the input images in this study is represented in Figure 17, and we can see that certain features have a regular gray gradient shape over time.

Based on observations of several input images, it is reasonable to expect that the filter in the convolution layer will act as an important factor in extracting the features of the image. It is also expected that the label overlapping ratio, which shows how many past datapoints are used to encode current labels, will play an important role in improving FGCC accuracy. This is the reason why we performed a sensitivity analysis of the two major parameters for finding an optimal combination of these two parameters.

In this study, level average analysis using the Taguchi method was applied to examine the individual sensitivity of all parameters in terms of width, height of the convolution filter and label overlapping ratio; every combination was evaluated in terms of the training, test, and validation accuracies, which are the so-called the-larger-the-better indices. As a result, the three 3-level parameters to be examined are shown in Table 2, and an orthogonal array table of *L*_9_(3^3^) is also shown in Table 3 with results of the training, test and validation accuracies by parameter combination.

**Figure 18:**
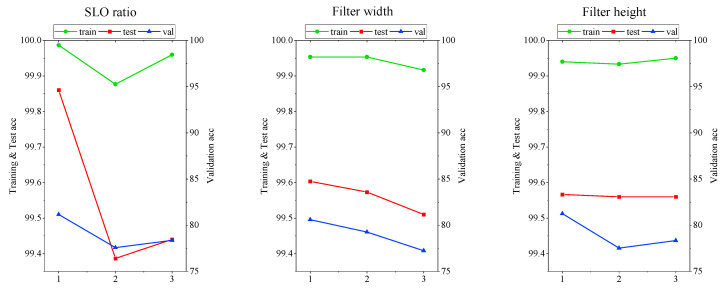
Results of the level average analysis of three major hyper parameters in terms of training, test, and validation accuracy.

60% and 40% in the entire dataset of 360,000 were used for training and test, respectively. And 1506 validation datapoints were predicted with each model obtained by a combination of orthogonal array tables. After performing learning along the orthogonal array table, the level average analysis, as in Figure 18, confirmed that the SLO ratio was the parameter that had the greatest effect on learning and validation accuracy, and that all three parameters showed the highest accuracy at 1st combination.

As a result of the level average analysis, the best combination of the parameter levels was confirmed as being the first combination in training with a batch size of 4000 and 10,000 epochs. The feature map created by the filter for each layer at this combination is presented in Appendix E.

**Figure 19:**
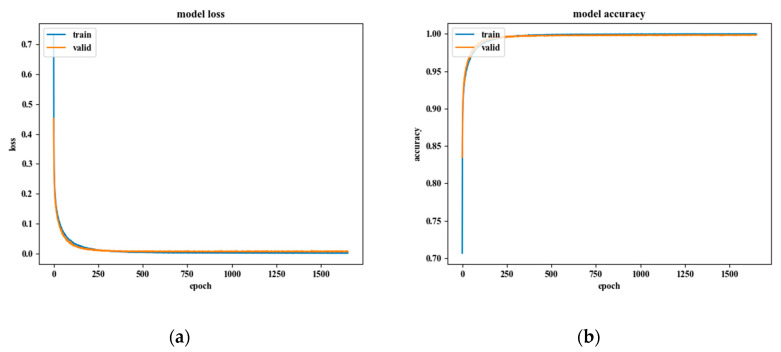
Results of the CNN model training: (**a**) model loss, (**b**) model accuracy.

Figure 19a indicates the loss change of the model: the loss change for the training-set was 0.000954 and the test-set was 0.005478. In addition, Figure 19b represents the accuracy change of the model, with a value of 0.9997 for the training-set and a value of 0.9984 for the test-set.

In addition, validation data was predicted through the trained model, and the four positions were classified with an average probability of 84.80%. Detailed accuracy results by label are shown in Table 4.

## 5. Results and Discussion

In this study, the SLO method is proposed to accurately detect the foot–ground contact phases composed of three sub-phases, as well as the swing phase using only wearable motion sensors attached to the foot and shank. We succeeded in developing a CNN model with a learning and test accuracy of 99.8% or more and confirmed that its validation accuracy was close to 85%.

Especially, whereas many previous studies did not consider overlapping labels in sliding window-based time-series data capture, this study shows that FGCC via CNN at a rate of 100 Hz can be realized with the proposed SLO method. Studies without labeling overlap have significant disadvantages in terms of real-time monitoring and reliability as they can only be used in limited situations. In this study, to overcome these shortcomings, a sliding window method was applied, which opens wider fields of applications and research.

However, more diverse studies are needed to verify the data augmentation method utilized in this study. Although the method of applying noise generated by sensors was sufficiently useful, there was a limitation in that the disturbance or deformation generated while walking could not be applied. In future studies, it is necessary to investigate various methods for improving classification accuracy in the real-world through sensor fusion of EMG [35], IMU, etc. as well as the data augmentation.

## Figures and Tables

**Table 1:** Input parameters and hyper parameters of CNN model.

Input Parameter		CNN Model Parameter					
SLO width	Image Shape	Pooling method	Layer no.	Filter size	Drop-out rate	Stride width	Activation function
14	22×14	Average-Pooling	3	m×n	0.3	1	ReLU

**Table 2:** 3-level parameter table including SLO ratio and size of the convolution filter.

Level	SLO Ratio [%]	Filter Width	Filter Height
1	30(4)	3	3
2	50(7)	5	5
3	70(10)	7	7

**Table 3:** Orthogonal array of *L*_9_(3^3^) with training, test and validation accuracies by combination.

No.	SLO Ratio	Filter Width	Filter Height	Train Acc [%]	Test Acc [%]	Val Acc [%]
1	1	1	1	99.97	99.84	84.80
2	1	2	2	100	99.88	79.40
3	1	3	3	99.99	99.86	79.29
4	2	1	2	99.90	99.48	78.23
5	2	2	3	99.87	99.33	76.96
6	2	3	1	99.86	99.35	77.55
7	3	1	3	99.99	99.49	78.77
8	3	2	1	99.99	99.51	81.41
9	3	3	2	99.90	99.32	74.92

**Table 4:** Result of experimental validation set data [%].

	SW	HS	FC	HO	Total
Accuracy	82.27	81.61	82.12	93.18	84.80

**Figure A1:**
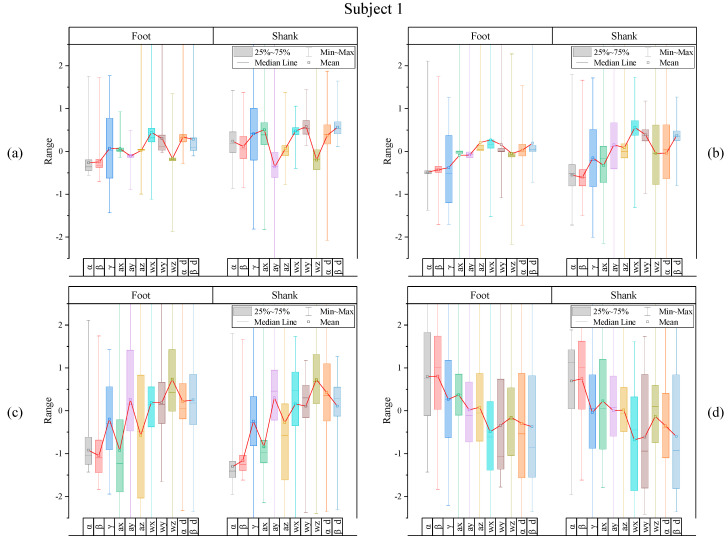
(**a**) label 2, (**b**) label 3, (**c**) label 4, (**d**) label 5.

**Figure A2:**
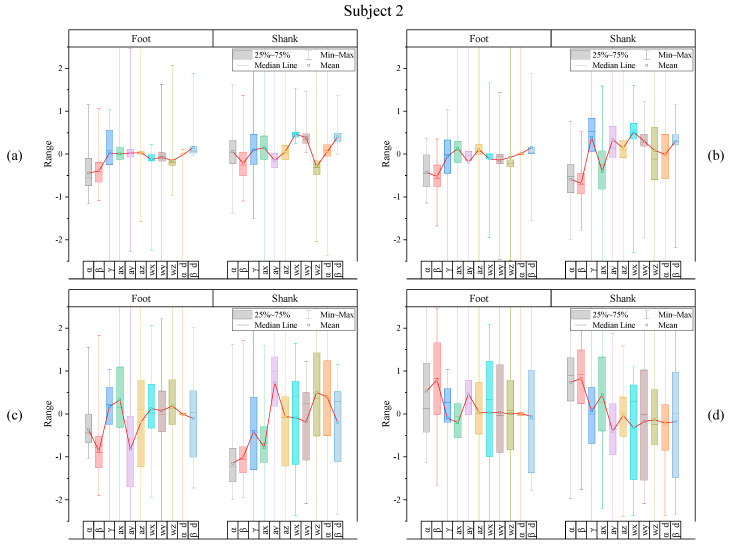
(**a**) label 2, (**b**) label 3, (**c**) label 4, (**d**) label 5.

**Figure A3:**
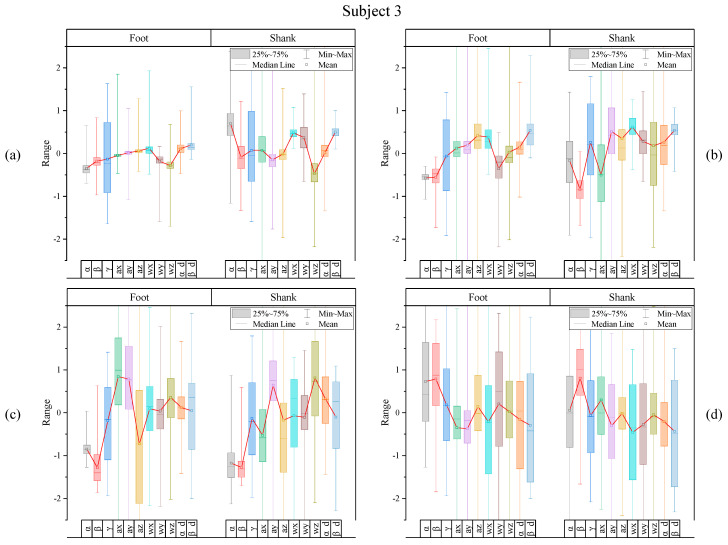
(**a**) label 2, (**b**) label 3, (**c**) label 4, (**d**) label 5.

**Figure A4:**
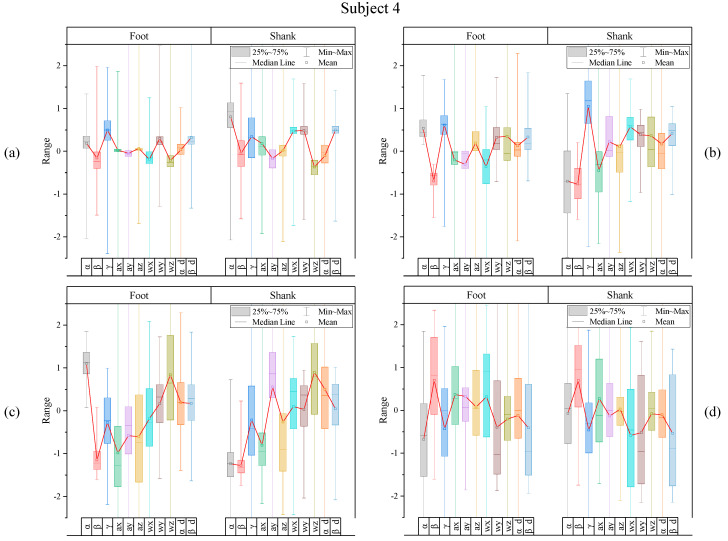
(**a**) label 2, (**b**) label 3, (**c**) label 4, (**d**) label 5.

**Figure A5:**
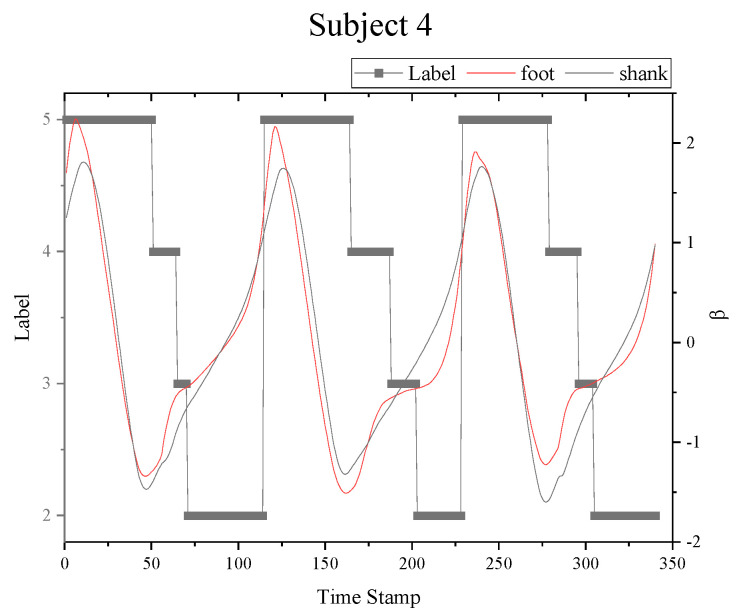
Pitch angles of foot and shank of subject 4.

**Figure A6:**
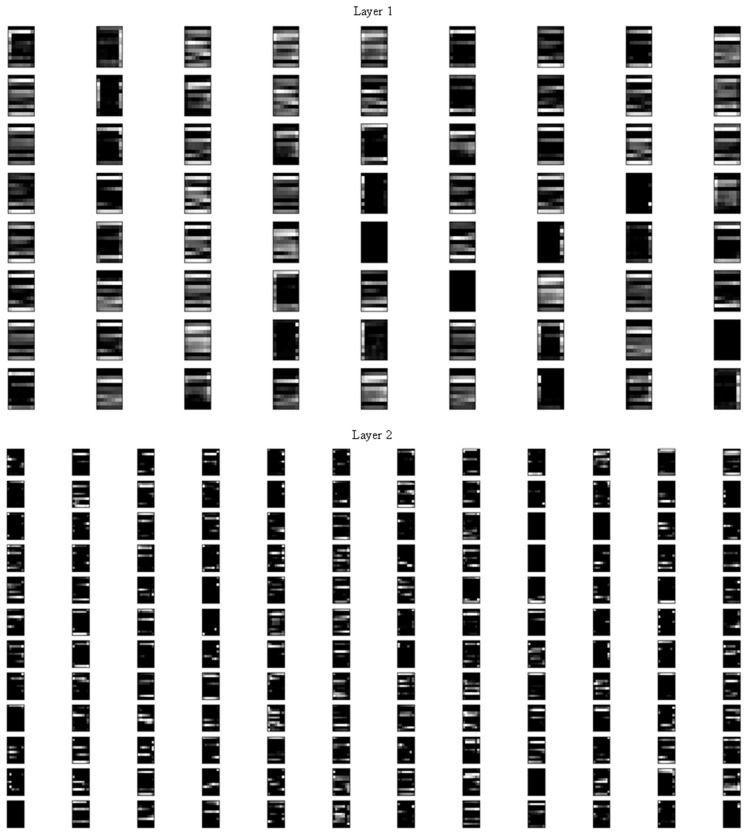

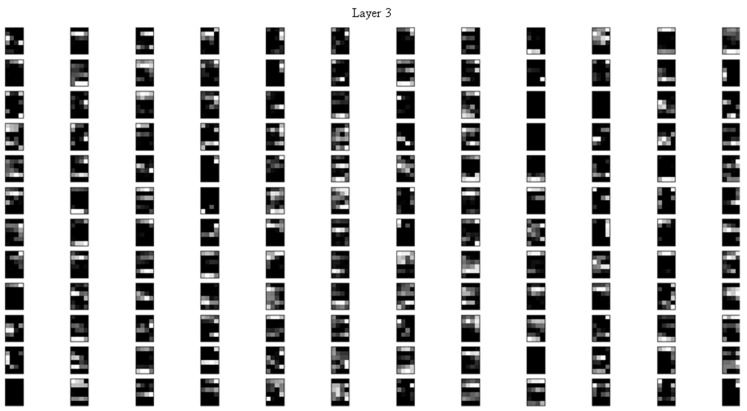
Resultant feature map obtained with the 1st hyper parameter combination in Table 3.

## Appendix A

**Algorithm A1** Pseudocode of four sub-phase labeling process
1: Interrupt Service Routine (every 10 ms)
2: **if** (read FSR0 > 10 kg_f_)
3: FSR0 = True
4: **else**
5: FSR0 = False
6: **if** (read FSR1 > 10 kg_f_)
7: FSR1 = True
8: **else**
9: FSR1 = False
10: **if** (read FSR2 > 10 kg_f_)
11: FSR2 = True
12: **else**
13: FSR2 = False
14: **if** (FSR0 and FSR1 = False **and** FSR2 = True)
15: Output = Heel Strike
16: **else if** (FSR0 = False **and** FSR1 = True **and** FSR2
17: = True) **or** (FSR0 = True **and** FSR1 = False
18: **and** FSR2 = True)
19: Output = Full Contact
20: **else if**(FSR0 = True **and** FSR1 = True **and** FSR2
21: **=** False)
22: Output = Heel off
23: **else if**(FSR0 = True **and** FSR1 = False **and**
24: FSR2 **=** False)
25: Output = Toe off

## Appendix B

**Table A1:** Means, standard deviations of every feature of each label.

Label 2											
	Orientation			Acceleration			Angular Velocity			Rate of Euler Angle	
	Yaw(α)	Pitch(β)	Roll(γ)	$a_{x}$	$a_{y}$	$a_{z}$	$w_{x}$	$w_{y}$	$w_{z}$	$\dot{\alpha }$	$\dot{\beta }$
Foot	−0.1551 (±0.4065)	−0.0377 (±0.3778)	−0.1357 (±1.0435)	−0.0044 (±0.6404)	0.0074 (±0.2723)	0.0367 (±0.2364)	0.0494 (±0.4883)	0.0514 (±0.6035)	−0.2059 (±0.5320)	−0.00019 (±0.6639)	0.1616 (±0.3891)
Shank	0.3824 (±0.5310)	0.2539 (±0.4975)	−0.0946 (±1.0713)	0.4032 (±0.6129)	−0.3422 (±0.4613)	0.0198 (±0.3701)	0.3205 (±0.4281)	0.3953 (±0.4091)	−0.2978 (±0.4173)	0.1115 (±0.4251)	0.4286 (±0.2765)
**Label 3**											
	**Orientation**			**Acceleration**			**Angular Velocity**			**Rate of Euler Angle**	
	Yaw(α)	Pitch(β)	Roll(γ)	$a_{x}$	$a_{y}$	$a_{z}$	$w_{x}$	$w_{y}$	$w_{z}$	$\dot{\alpha }$	$\dot{\beta }$
Foot	−0.3302 (±0.4323)	−0.2600 (±0.3925)	0.0593 (±0.8412)	−0.0069 (±0.8265)	−0.0069 (±0.6012)	0.2146 (±0.5781)	0.0421 (±0.5644)	−0.0461 (±0.4672)	−0.0828 (±0.6505)	0.0063 (±0.8427)	0.1086 (±0.4537)
Shank	−0.2768 (±0.5474)	−0.4322 (±0.4090)	0.2467 (±0.8486)	−0.2761 (±0.8104)	0.2586 (±0.8352)	0.0909 (±0.7499)	0.3244 (±0.5664)	0.2431 (±0.3592)	−0.1093 (±1.0977)	−0.0776 (±0.9521)	0.2585 (±0.3217)
**Label 4**											
	**Orientation**			**Acceleration**			**Angular Velocity**			**Rate of Euler Angle**	
	Yaw(α)	Pitch(β)	Roll(γ)	$a_{x}$	$a_{y}$	$a_{z}$	$w_{x}$	$w_{y}$	$w_{z}$	$\dot{\alpha }$	$\dot{\beta }$
Foot	−0.5414 (±0.6849)	−0.9097 (±0.6816)	0.0868 (±1.0554)	0.0475 (±1.4317)	−0.0921 (±1.5240)	−0.4572 (±1.5838)	0.1292 (±0.9869)	0.0885 (±0.8901)	0.4093 (±1.0212)	−0.0021 (±0.9594)	0.0802 (±1.0908)
Shank	−1.0112 (±0.6641)	−1.0129 (±0.5170)	−0.1106 (±0.9957)	−0.6221 (±0.8928)	0.5041 (±1.0838)	−0.1153 (±1.8441)	−0.0473 (±1.1490)	−0.1010 (±0.8285)	0.6609 (±1.1596)	0.3421 (±1.0326)	−0.1097 (±0.9633)
**Label 5**											
	**Orientation**			**Acceleration**			**Angular Elocity**			**Rate of Euler Angle**	
	Yaw(α)	Pitch(β)	Roll(γ)	$a_{x}$	$a_{y}$	$a_{z}$		$w_{y}$	$w_{z}$	$\dot{\alpha }$	$\dot{\beta }$
Foot	1.0371 (±1.3766)	1.1429 (±1.1608)	0.0018 (±1.0502)	−0.0273 (±1.0757)	0.0821 (±1.2921)	0.0955 (±1.1537)	−0.2158 (±1.6274)	−0.0773 (±1.6817)	−0.0241 (±1.5070)	−0.0058 (±1.4361)	−0.3793 (±1.6018)
Shank	0.7715 (±1.1886)	1.1079 (±1.0357)	−0.0841 (±1.0382)	0.4077 (±1.2366)	−0.3574 (±1.2485)	−0.0321 (±0.6482)	−0.7134 (±1.3111)	−0.6467 (±1.6110)	−0.0981 (±0.9490)	−0.3254 (±1.3263)	−0.6997 (±1.5509)

## Appendix C

Box plots of all features of every subject participated in this study after standardization process.

## Appendix D

**Algorithm A2** Pseudocode for assigning the representative label to a single window composed of the multiple labels
1: **Function** SLO (Entire labeled data set M (${\mathbb{R}}^{n\times 22}$),
2: SLO_width=14, SLO_pitch=1, SLO_ratio=N)
3: (Rotate 90 degrees counterclockwise to create
4: 22×14 images)
5: **rotate** M CCW 90 ← M(23×n)
6: **for** i=1:SLO_pitch:(size(M,2)-SLO_width)
7: slo_image(:,:,i) = M([i:slo_width+(i-1)],[2:23])
8: → Save the 22×14 image in three dimensions
9: slo_label(1,:,i) = M([i:slo_width+(i-1)],1)
10: → Save the 1×14 label in three dimensions
11: **end**
12: **for** i=1:1:size(slo_image,3)
13: **if** (slo_label(1,14-(N-1):14,i)==5)
14: **save** mat2gray(slo_image(i)) to the folder "L_5"
15: **else if** (slo_label(1,14-(N-1):14,i)==4)
16: **save** mat2gray(slo_image(i)) to the folder "L_4"
17: **else if** (slo_label(1,14-(N-1):14,i)==3)
18: **save** mat2gray(slo_image(i)) to the folder "L_3"
19: **else if** (slo_label(1,14-(N-1):14,i)==2)
20: **save** mat2gray(slo_image(i)) to the folder "L_2"
21: **end**
22: **end**
